# Bibliographical Notices

**Published:** 1846-11

**Authors:** 


### BIBLIOGRAPHICAL NOTICES.

*Elements of Pathological Anatomy; illustrated by colored engravings, and two hundred and fifty wood cuts; by* Samuel D. Gross, M. D. *Prof. of Surgery in the Medical Institute of Louisville, &c., &c. Second edition, thoroughly revised, and greatly enlarged.* Phila. Ed. Barrington and Geo. D. Haswell, 1845; p.p. 813, 8vo.

The above is the title of a work creditable alike to the zeal, the industry and the ability of the author, and to American medical literature. Its intrinsic merits, not less than its *native* character, entitle it to a place in the library of every American practitioner.

It would be entirely supererogatory to advocate the importance of the branch of Medical Science to which it relates. No one probably will deny the necessity of a thorough acquaintance with pathological anatomy as an essential element of medical knowledge, without which it is impossible to deserve the character of a scientific and successful practitioner; yet it may be doubted if it receive generally a degree of attention commensurate with its importance. It is much too frequently neglected, both as a subject of reading, and as involving the duty of making, as often as circumstances will permit, autopsical examinations. These two methods of its prosecution should be pursued conjunctively. Let the practitioner keep himself informed, by means of books, of the investigations of others, and let him, also, verify facts, and, if possible, add to them, and extend them by his own observations. In this way he will, at least, have the satisfaction of keeping pace with the advancement of science, and, perhaps, the additional enjoyment of aiding in its onward progress.

As we have but recently received this extensive work, we cannot profess to have given it an entire perusal. We have, however, examined it with considerable attention, and feel prepared to say that it is comprehensive in its scope, and, in the main, appears to present an accurate view of the branch of science of which it treats. The author gives proofs of extensive and careful erudition in availing himself of the labors of those who have contributed to the present advanced position of pathological anatomy, and his work, also, in the amount and character of original observations, affords abundant evidence of industry and talent in developing and confirming facts. A great merit of the work consists in its eminently practical character. Notwithstanding its size, little space is bestowed upon subjects of merely speculative interest, but the useful every where appears as the actuating motive and aim in its preparation.

By this general commendation we do not, by any means, mean to imply that the work is faultless. Were we to enter upon an analysis of its contents, (which its nature and magnitude preclude,) we should doubtless find room for the exercise of criticism. We will not close this notice without brief reference to one error (as we regard it) which in our apprehension is a serious blemish, and is calculated, in so far as it may affect the views of students of medicine and practitioners, to inculcate a pernicious doctrine. We have observed in most of the reviews of the work which we have met with, that the point to which we allude has furnished occasion for disagreement between the reviewer and author. The point, or rather points to which we refer are contained in the following quotation: "It may be assumed as a general position, liable to few exceptions, that all organic diseases whatever be their seat or extent, are the result of inflammatory action either of an acute or chronic kind. The second proposition that may be stated is, that every inflammation, irritation, or morbid action is originally of a local nature; that is to say, it always makes its impression in the first instance upon some particular part, texture or organ." (p. 22.)

Before stating these propositions the author premises the signification which he intends to attach to the term *organic*. He says, "by pathological anatomists the word is generally employed to denote some permanent change in the textures of an organ; but in the present sense, I would not only include under it all such lesions, but also every temporary alteration which the tissues experience when in a state of disease. The term organic will thus have a wider latitude; and as expressing the same thing, we shall often have occasion to use the word structural. If this acceptation be adopted, it may perhaps be doubted whether, under any circumstances, there can, strictly speaking, be a functional disease, or in other terms, a mere aberration of the physiological state of a part without some change in its anatomical elements. The question, at all events, is not settled." (p. 22.) Assuredly the question is not settled, and, on this very account, we think this change in the signification of terms, which the author sees fit to adopt, is unauthorized and objectionable. The terms *organic* and structural are used to distinguish lesions in which there is a palpable, demonstrable change in organic elements, from other affections in which no such sensible appearances are discoverable. Employed in this way these terms have definite significations, as well as practical utility; and, besides, conventionality and long usage have sanctioned their use in this sense. We cannot, therefore, see any intrinsic propriety in giving them a latitude which shall embrace all diseased actions, on the ground that it is a matter of question that a change in organic elements may exist in all instances. It will be time enough to make this alteration when the fact has been demonstratively settled; as it is, the terms with this comprehensive meaning loose all significancy, and are quite superfluous—in fact expressing no more than the general term disease.

This definition is to be considered in connection with the passages previously quoted. It will be seen that he, first, assumes that all diseases are organic; second, that, with a few exceptions, all diseases are inflammatory; and, third, that all diseases are primarily local. Now, we do not quarrel with Prof. Gross for holding these doctrines, but, in so far as our opinion of the doctrines are concerned, we cannot express ourselves too strongly in opposition to them. We believe that the prevalence of these doctrines has been of incalculable injury as the basis of medical practice. The conviction forces itself upon our mind that the habit of seeing inflammation on all occasions as the only fundamental principle of diseases, has been attended with destructive consequences, which we dare not estimate, lest we do injustice to a profession which claims our respect and veneration. Every observer must perceive that in this respect medicine has changed within the last ten years, and that the change is still progressive; and in our view this change is indicative of progress, and constitutes at this moment a characteristic, and the most important feature of medical science. Our own opinion, which we express thus freely, is of course of small consequence; but with regard to the positions assumed by Prof. G., we believe we are warranted in saying that they are in opposition to the opinions entertained by the larger portion of the highest living authorities. This, we think, he was bound to have stated more fully, so as not to lead the student to infer that from being found in a work intended to have a standard currency, they express doctrines which are generally accredited. We can hardly be mistaken in supposing that Prof. G. will find but few, at the present day, who will unite with him in referring the various disorders included in the neuroses, the heterologous productions and other aberrations of nutrition, the different cachexiæ, disorders of assimilation, secretion, &c., &c., in all, or nearly all cases, to local inflammation as the primary fundamental form of disease. Nor is he sustained by the general sentiment of the profession in saying "that the time cannot be far off, when the term *fever* must be entirely discarded from our books, and diseases named from the tissues they implicate."

In this criticism we shall not be accused of inconsistency in our previous commendation of the work; still less, of doing injustice to our sense of the talents and erudition of the author. While we think the pathology of the work should be repudiated, we do not find fault with it as, strictly speaking, a work on pathological anatomy, but, on the other hand, in so far, we would advocate in strong terms its claims to confidence and patronage.

The plates are numerous, and in comparison with those of American works generally, are superior. The style is always direct, but in aiming at an agreeable ease and familiarity, the author exposes himself to the criticism of being careless in his composition. Elegance of diction evidently was not a predominant object, and perhaps was considered undesirable. It is not an uncommon sentiment that scientific works are equally, if not more acceptable, when written without regard to elegance and classical propriety of diction. Tastes on this, as on all other matters will differ; but in our estimation no subject loses in force or attractiveness by being judiciously associated with rhetorical graces. T. and M. Butler will receive orders.

---

*Lectures on the Theory and Practice of Physic, by* John Bell, M. D., etc., *and* Wm. Stokes, M. D., etc. *Third edition, enlarged and improved. In two volumes, 8vo.* Philadelphia, Ed. Barrington and Geo. D. Haswell. 1845.

For the value of this work on practical medicine, the names of the two distinguished medical gentlemen associated in authorship afford a sufficient guaranty. Of the high consideration in which it is held by the profession, the fact of its being generally adopted among the text-books of the medical schools, and, the rapid sales of the previous editions, are sufficient evidence. The third edition has been enlarged to the extent of three hundred pages, besides being otherwise improved, to adapt it to the present condition of our ever progressing science. In some of his opinions the American author is peculiar. He contends, for example, for the non-existence of a specific miasm as the causative agent of intermittents. But our profession admits of the largest liberty in the formation and discussion of different opinions and doctrines. The reader will find some cogent arguments for the peculiar position just referred to, although few will experience conviction of their sufficiency to sustain it. The work may be recommended as a comprehensive and faithful digest of the Theory and Practice of Medicine as taught by the best masters of the present day. T. & M. Butler will receive orders.

---

*The American Journal and Library of Dental Science.* Baltimore.—We have omitted notice of this periodical hitherto, not from any want of appreciation of its character, but through inadvertency. It is a quarterly of 120 pages, and, in addition, the last number contains 80 pages of a standard treatise on Dentistry. Its editors are Chapin A. Harris, M. D. and Edwin J. Dunning. It is conducted with ability and vigor, and must prove acceptable to every dental practitioner, as, indeed, also, to the general practitioner of Medicine. Terms, $5 00 per annum.

The *Baltimore College of Dental Surgery* appears to be highly flourishing. A commodious college edifice has recently been erected; and four professorhips established and filled. The lectures will commence in Nov., and continue sixteen weeks. The efforts which are being made to place the science of den'istry on a proper scientific basis, and to separate the competent operator from the ignorant pretender, are honorable to those who are actively engaged in the undertaking, and should be appreciated by the public whose interests are thereby to be promoted.

---

*The Diagnosis, Pathology and Treatment of diseases of the Chest. By* W. W. Gerhard, M. D., *Lecturer on Clin. Med. to the University of Pennsylvania, &c. Second edition, revised and enlarged.* Philadelphia, Ed. Barrington & George D. Haswell, 1846. 8*vo. pp.* 188.

We have already taken occasion to recommend this as the very best of the manuals which have fallen under our observation. To the student or practitioner, who wishes to become acquainted with auscultation and percussion, together with the most approved principles of the pathology and the treatment of the diseases of the chest—we would say, by all means study this volume. It presents all the important practical facts compactly, lucidly, and with simplicity. The present edition is in several respects improved. T. & M. Butler will receive orders.

